# Promising Modulatory Effects of Cenicriviroc on the Progression of Mouse Colorectal Cancer through Inhibition of CCR2_CCL2 Signaling Pathway

**DOI:** 10.1155/2023/5993866

**Published:** 2023-06-06

**Authors:** Mina Eslami, Farid Azizi Jalilian, Rezvan Najafi, Ali Mahdavinezhad, Razieh Amini

**Affiliations:** Molecular Medicine Research Center, Hamadan University of Medical Sciences, Hamadan, Iran

## Abstract

The study was designed to assay the efficacy of cenicriviroc (CVC) on the progression of mouse colorectal cancer by downregulation of CCR2_CCL2. In this study, CVC was used to inhibit the CCR2 receptor. Next, an MTT assay was performed to evaluate the cytotoxic effects of CVC on the CT26 cell line. CT26 cells were implanted subcutaneously in BALB/c mice. After tumor implantation, one group of animals received 20 mg/kg of CVC several times. The mRNA levels of CCR2, CCL2, VEGF, NF-*κ*B, c-Myc, vimentin, and IL33 were determined in the CT26 cell line and then tumor tissues (after 21 days), by qRT-PCR. Protein levels of the above-mentioned targets were determined by western blot and ELISA. Flow cytometry was performed to assess the changes in apoptosis. Tumor growth inhibition was measured on the 1st, 7th, and 21st days after the first treatment. In both cell line and tumor cells treated with CVC, expression levels of the markers of our interest in mRNA and protein levels were significantly reduced compared to controls. A significantly higher apoptotic index was observed in CVC-treated groups. The rates of tumor growth were significantly decreased on the 7th and 21st days after the first injection. To our knowledge, this was the first time that we demonstrated the promising effect of CVC on the development of CRC through inhibition of the CCR2_CCL2 signaling and its downstream biomarkers.

## 1. Introduction

Colorectal cancer (CRC) is known with a high prevalence rate. Many risk factors such as age, family history, and lifestyle including diet, physical activity, and smoking affect its occurrence [1]. CRC tumors can be surgically removed, but for the tumor in advanced stages, tumor metastasis and progression are important issues [2]. Several fundamental factors in the tumor microenvironment contribute to the development of cancer [3]. The cellular interactions in the tumor microenvironment can facilitate metastasis cascades which are often possible through the signaling of chemokines [4]. CCL2 is over-expressed in CRC, along with its main receptor CCR2 boosts the progression of cancer by promoting proliferation, survival, angiogenesis, migration, and invasion [5] and contributes to tumor metastasis [6].

Studies have shown that MDSCs (myeloid-derived suppressor cells) [7] and TAMs (tumor-associated macrophages) are attracted and accumulated in the tumor site by CCL2 [8]. TAMs and MDSCs are important cells in the development of cancer [5]. Studies have shown that TAMs promote cancer progression by increasing invasiveness and angiogenesis [9]. TAMs produce EGF (epidermal growth factor) which enhances the invasion and motility of cancer cells through increasing matrix degradation [10]. In addition, TAMs inhibit T cell proliferation and suppress the immune system by producing immunosuppressive molecules, which ultimately lead to the development of cancer [11, 12]. MDSCs expand strongly in some pathological conditions, including cancer. In addition, the activity of immune cells is regulated by these cells, and evidence has shown that MDSCs can cause tumor growth and cancer progression by inhibiting T cells that have antitumor activity [13].

In addition to the cases mentioned, CCL2 is produced by cancer cells, and TAMs and CAFs (cancer-associated fibroblast) can react with the CCR2 receptor expressed on the surface of cancer cells and cause its activation, which subsequently increases the proliferation, migration, and resistance of cancerous cells to apoptosis. In addition, CCL2 by binding to the CCR2 receptor on the surface of vascular endothelial cells can increase angiogenesis. This chemokine increases the attraction and recruitment of Tregs (T regulatory cells) in the tumor microenvironment, which, in turn, suppresses immunity and increases cancer progression [7].

According to these documents, the CCR2_CCL2 signaling pathway plays a key role in the development of cancer, which makes the CCR2 receptor an important and potential therapeutic target in cancer treatment [5].

Cenicriviroc (CVC) is a dual inhibitor of CCR2 and CCR5 receptors. This compound was initially used to treat individuals with HIV [14]. It has also recently shown promising results in limiting inflammation and fibrosis in liver fibrosis disease [15].

Considering the significant role of the CCR2 receptor in cancer, in this study, for the first time, we studied the effect of this compound in preventing the development of CRC in mouse models and cell lines through inhibition of the CCR2_CCL2 signaling pathway.

Herein, we examined the effects of CVC on the mRNA and protein levels of CCR2, CCL2, VEGF, NF-*κ*B, c-Myc, IL33, and vimentin, which are involved in the progression of cancer. VEGF is the key mediator of angiogenesis in cancer. Angiogenesis is necessary for the development of cancer, and therefore, VEGF can promote the development of cancer [16].

The expression of many genes effective in the proliferation, inflammation, migration, and survival of cancer cells is regulated by nuclear factor kappa B (NF-*κ*B), which is an important transcription factor. This factor can cause the development of CRC [17].

Many genes that are effective in cellular processes are regulated by c-Myc, which is an important transcription factor [18].

High expression of this factor has been observed in CRC. Similarly, some studies have shown a strong correlation between the positive expression of c-Myc and poor prognosis in patients with CRC [19].

IL33 is a member of the super family of interleukin 1 cytokines, which plays an important role in inflammation [20]. It can cause tumor progression, and other research has proven that IL33 can increase the invasion and migration of glioma cancer cells by up-regulating MMP2 and MMP9 through the ST2_NF-*κ*B signaling pathway [20]. Abnormal expression of IL33 has been reported in human CRC tissues. Also, studies have shown that IL33 can facilitate the proliferation of CRC cells through the ST2 receptor and NF-*κ*B signaling, which up-regulates the expression of COX2 and PGE2 [21].

Vimentin is one of the main components of the IF (intermediate filament) family that preserves cell integrity. Vimentin over-expression increases tumor growth, invasion, and poor prognosis in cancer. It is also considered an important marker for EMT [22]. Initiation and progression of metastasis are enhanced by EMT [23]. Over-expression of vimentin in some cancers including CRC has been reported [22].

Our study suggested that CVC might be a promising compound to reduce the progression of CRC through inhibition of the CCR2-CCL2 signaling pathway.

## 2. Material and Method

### 2.1. Material and Reagents

CVC (chemical formula: C41H52N4O4S) lyophilized powder (lot: 8721-25 mg-97%), dimethyl sulfoxide (DMSO), trypan blue, MTT powder (molecular weight: 335.42), acryl amide, bis-acrylamide, and tris base were prepared from Sigma-Aldrich; 1 mM stock of CVC (MW 696.96 g/mol) was prepared by adding DMSO; then, different concentrations of CVC ranging from 20 to 100 *μ*M were prepared from this stock. Gene MATRIX Universal purification kit (Poland, cat no. E3598) was used. The cDNA synthesis kit was prepared from the REVERTA-L RT kit (Moscow, Russia). SYBR Green QPCR Master Mix, DNA ladder 1 kb, and 100 bp (Thermo Fisher Scientific, USA) were used. The Bio Basic protein extraction kit was used.

TEMED, APS, and isopropanol were prepared from Merck (Germany). Antirabbit IgG H&L (HRP) (ab6721) secondary antibody, anti-CCR2 (ab203128), antivimentin (ab92547), anti-NF-*κ*B (ab16502), anti-c-Myc (ab32072), anti-IL-33 (ab187060), and anti-*β* actin-loading control antibodies (ab8227) were purchased from Abcam (Cambridge, AM, USA).

### 2.2. Cell Culture

The CT26 (mouse colon cancer) cell line was bought from the Pastor Institute of Iran (Tehran, Iran). Dulbecco's modified Eagle's medium (DMEM) low glucose (Gibco, CA, USA), containing 10% fetal bovine serum (Gibco, CA, USA) and 1% penicillin-streptomycin (Gibco, CA, USA), was used for cell culture, and then, the cells were incubated in a 37 C incubator containing 5% CO_2_.

### 2.3. MTT Assay

MTT assay was performed to determine IC_50_. First, 1 × 10^4^ cells were seeded per well into a 96-well plate (triplicates). Then, different concentrations of CVC (20–100 *μ*M) were prepared and remained overnight; afterward, the cells were treated with different concentrations of CVC. After 24, 48, and 72 hours of incubation, 10 *μ*l of MTT (3-(4,5-dimethylthiazol-2-yl)-2,5-diphenyl tetrazolium bromide) reagent was added to each well, and after 4 hours of incubation at 37°C, the medium was discarded, and after that, 100 *μ*l of DMSO was added. Finally, the absorbance of solubilized formazan at 570 nm was determined with a multiwell plate reader.

### 2.4. Treatment of CT26 Cells with CVC

After the determination of IC_50_ (40 *μ*M), this concentration of the compound was used to treat the cells, and then, the conditioned media were discarded, and the complete cell culture medium was added. After 24, 48, and 72 hours of incubation, trypsin was used to separate the cells, and subsequent analyses were performed.

### 2.5. RNA Isolation and QRT-PCR

RNA extraction was performed from CT26 cells using the RNA extraction kit. Then, RNA quality was determined by using gel electrophoresis, and ribosomal RNA bands (18 S and 28 S) were observed on the gel. Then, RNA concentration and purity were measured by NanoDrop (Thermo Fisher Scientific, Waltham, MA) spectrophotometer. After the synthesis of cDNA from RNA, the expression of the target genes was evaluated using the real-time PCR technique. Real-time PCR was performed with 1 *μ*l cDNA, 3.6 *μ*l H2O, 5 *μ*l SYBR Green qPCR Master Mix, and 0.2 *μ*l of specific primers in the Roche Light Cycler machine (Roche Diagnostics). At this stage, the 18 s rRNA gene was used as a reference gene. Finally, the relative expression of the genes was obtained by using the 2^−ΔΔCT^ method. In Table 1, the primer sequences are shown.

### 2.6. ELISA

To quantitate the soluble form of CCL2 and VEGF proteins in the conditioned medium of the CT26 cells, a commercial ELISA kit (R&D Systems) was used, and according to the kit protocol, we assessed the level of these proteins. At an absorbance of 450 nm, the light absorption of the standard and samples were read.

### 2.7. Western Blot Analysis

First, by using trypsin, a cell suspension with a concentration of 1 × 10^7^ CT26 cells was prepared. Then, the cells were washed using PBS and then lysed using RIPA buffer (Santa Cruz, USA). The lysates were removed by centrifugation at 14,000 rpm for 20 min at 4°C. After that, the Bradford method was used to determine the protein concentration. The lysates were mixed with an equal volume of 2X Laemmli sample buffer, and then, the lysates were exposed to SDS-PAGE (50 *μ*g) and subsequently transferred to the PVDF membranes. After transferring, 5% BSA (Sigma-Aldrich) was used for membrane blocking. After blocking, incubation with primary antibodies (1 : 1000) was done, and then, the membranes were washed with TBST. Subsequently, incubation with goat anti-rabbit IgG H&L (HRP) secondary antibody (1 : 4000) was done, and after that, chemiluminescence detection kit (ECL, Amersham, USA) was used to visualize protein bands, and finally, the bands were quantified by using Image J software.

### 2.8. Flow Cytometry

To evaluate the number of apoptotic cells, the flow cytometry technique was used. After seeding CT26 cells (1 × 10^6^) into a 6-well plate and treating the cells with CVC, the cells were collected and washed, and then, according to the steps of the kit (Mab-Tag, Germany), the cells were stained with Annexin V and propidium iodide (PI). Finally, the amount of apoptosis in the treated and control cells was determined by using Attune NxT acoustic focusing cytometer (Life Technology, USA).

### 2.9. Animal Experiment

For animal experiments, BALB/c mice, male, 8 weeks old (25 gr), were bought from Pastor Institute of Iran (*n* = 10). Mice were fed with a normal mice pellet and water and maintained in suitable conditions including temperature (23°C ± 3°C), humidity (50 ± 10%), and 12 h light and dark cycle [24]. CT26 cells (400000) were injected subcutaneously to induce colorectal cancer tumors in mice. After 10 days, the mice were ready to receive treatment. These mice were categorized into two groups: the treated group (*n* = 5) and the control group (*n* = 5), and then, the treated mice were treated with 20 mg/kg of CVC three times a week for three weeks by intraperitoneal injection [15]. Control mice were injected with PBS. After 21 days, an anesthesia procedure was performed by using a ketamine/xylazine recipe and intraperitoneal injection of ketamine (100 mg/kg) plus xylazine (10 to 5 mg/kg), and later, tumor tissue in each group was removed and processed for the experiments. For this study, the Local Ethics Committee approval was obtained (IR.UMSHA.REC.1401.048).

### 2.10. Tumor Size Measurement

Tumor size in two groups was measured by a caliper. Tumor imaging and measurement were performed on days 0-7-21 after the first injection.

### 2.11. Real-Time PCR for Animal Tissues

After 21 days, tissues were collected, and in the next step, RNA extraction was done using the Universal RNA purification kit. Then, cDNA synthesis was performed. Finally, a real-time PCR was performed to determine the changes in gene expression.

### 2.12. Statistical Analysis

Graph Pad Prism 8.0 software was used to analyze the data. All the data were presented as a mean ± standard error of the mean. Analysis of variance (One-way ANOVA) was used to compare the data. A *p* value <0.05 was considered statistically significant.

## 3. Results

### 3.1. Treatment with CVC Reduced Cell Viability on CT26 Cells

**Figure 1:**
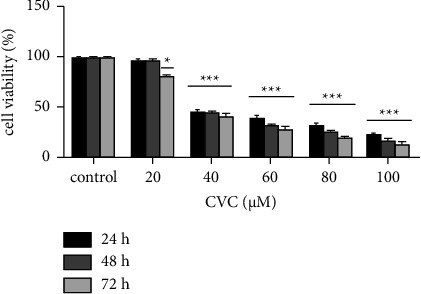
The effect of CVC on CT26 cell viability. CT26 cells were treated with different concentrations of CVC at 24, 48, and 72 h MTT assay was performed to determine the cell viability. The optimum concentration of CVC in this assay was 40 *μ*M. ^*∗*^*p* < 0.05, ^*∗∗*^*p* < 0.01, and ^*∗∗∗*^*p* < 0.001 vs. the control (*n* = 3).

MTT assay was performed to evaluate the cytotoxic effects of CVC on CT26 cell viability. Different concentrations of CVC (20-40-60-80-100 *μ*M) were prepared, and IC_50_ was determined (40 *μ*M). Finally, this IC_50_ concentration was used for further experiments (Figure 1).

### 3.2. Treatment with CVC Reduced the CCR2, CCL2, VEGF, Vimentin, c-Myc, NF-*κ*B, and IL33 mRNA Levels in CT26 Cells

**Figure 2:**
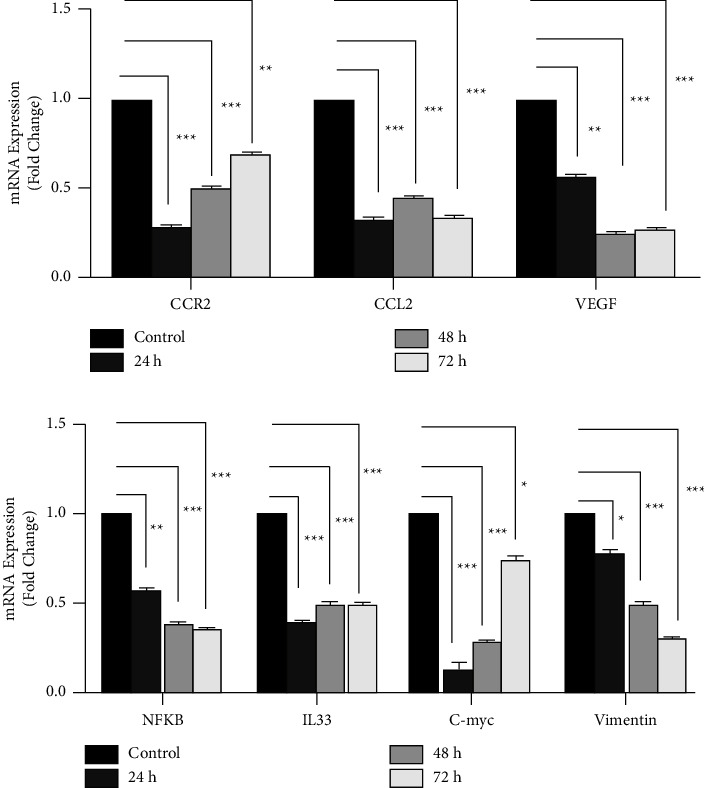
The effect of CVC on the expression of CCR2, CCL2, VEGF, NF-*κ*B, c-myc, vimentin, and IL33 by qRT-PCR. (a) and (b) The mRNA level of CCR2, CCL2, VEGF, NF-*κ*B, c-myc, vimentin and IL33 in CVC-treated cells significantly decreased in all-time points 24, 48, and 72 h. Mean ± SEM. ^*∗*^*p* < 0.05, ^*∗∗*^*p* < 0.01 and ^*∗∗∗*^*p* < 0.001 vs. the control (*n* = 2).

As shown in Figure 2(a), treatment with CVC in CT26 cells dramatically decreased the expression of CCR2, CCL2, and VEGF mRNA levels compared to the control groups in all times points: CCR2 (72% reduction for 24 h, 50% for 48 h, and 31% for 72 h), CCL2 (67% reduction for 24 h, 55% for 48 h, and 66% for 72 h), and VEGF (43% for 24 h, 75% for 48 h, and 73% for 72 h). Also, results showed a significant reduction in mRNA levels of vimentin (22% reduction for 24 h, 50% for 48 h, and 70% for 72 h), c-Myc (90% reduction for 24 h, 71% for 48 h, and 25% for 72 h), NF-*κ*B (42% reduction for 24 h, 61% for 48 h, and 64% for 72 h), and IL33 (60% reduction for 24 h, 50% for 48 h, and 72 h) in all treated time points (Figure 2(b)).

These data showed that CVC could expressively diminish the expression of CCR2, CCL2, VEGF, vimentin, c-Myc, NF-*κ*B, and IL33 genes in all treatment times.

### 3.3. Treatment with CVC Decreased CCR2, CCL2, VEGF, Vimentin, c-Myc, NF-*κ*B, and IL33 Protein Level

**Figure 3:**
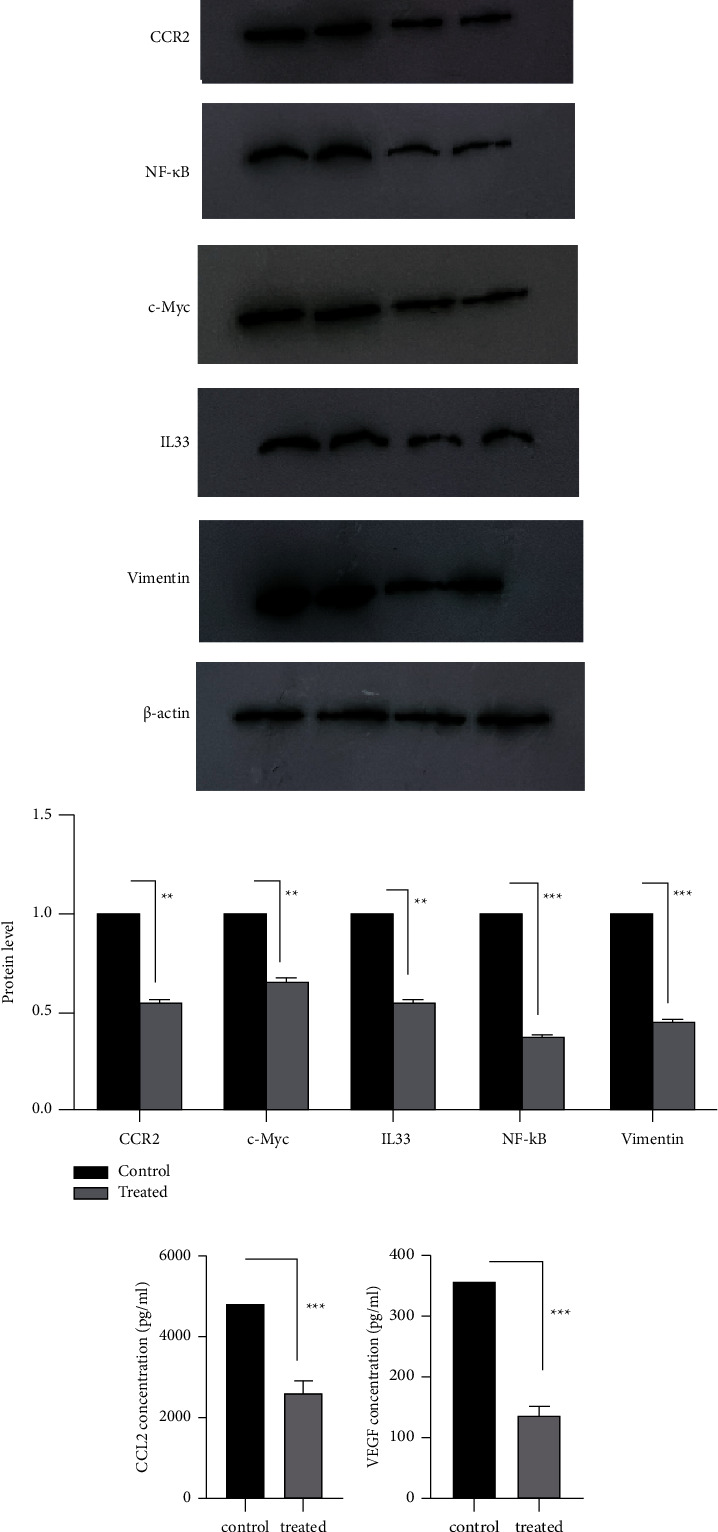
The effect of CVC on the level of CCR2, CCL2, VEGF, NF-*κ*B, c-myc, vimentin, and IL33 proteins by western blotting and ELISA. (a) The results obtained from western blot analysis showed that the level of CCR2, NF-*κ*B, c-myc, vimentin, and IL33 proteins was dramatically reduced in treated cells versus nontreated cells. *β*-actin was used as a reference protein. (b) VEGF and CCL2 protein levels in the conditioned medium of the treated cells versus nontreated cells were considerably reduced in treated cells assessed by ELISA. Mean ± SEM. ^*∗∗*^*p* < 0.01 and ^*∗∗∗*^*p* < 0.001 vs. the control (*n* = 2).

To evaluate the level of CCR2, vimentin, c-Myc, NF-*κ*B, and IL33 proteins, the western blotting technique was performed. Based on the results of QRT-PCR and mRNA level of the genes of interest in all time points, the time point of 48 h was selected for evaluation of protein level. The results obtained from this experiment showed that the amount of these proteins in the treated cells had a major reduction compared to the untreated cells (45% reduction for CCR2, 62% for NF-*κ*B, 34% for c-Myc, 55% for vimentin, and 44% for IL33), at the optimum time (Figure 3(a)). To assess the changes in the soluble form of CCL2 and VEGF proteins in the conditioned medium, ELISA was performed at the optimum time point of 48 h. The results obtained from this evaluation showed a considerable reduction in the level of CCL2 and VEGF proteins in the conditioned medium of the treated cells compared with the control (45.34% reduction for CCL2 and 60.94% for VEGF) (Figure 3(b)). The changes in protein levels confirmed the findings of our targets in mRNA levels.

### 3.4. Treatment with CVC Increased Apoptosis in CT26 Cells

**Figure 4:**
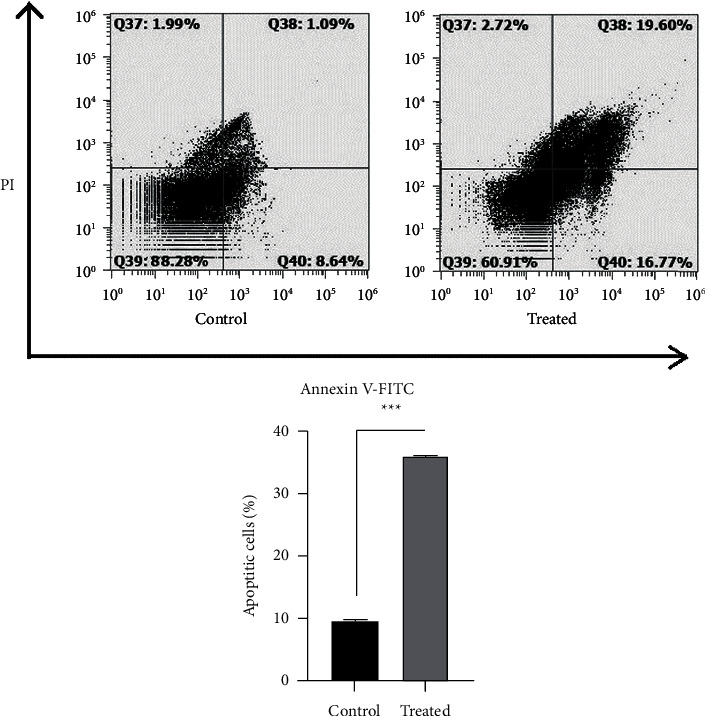
The effect of CVC on apoptosis of CT26 cells. CVC could promote apoptosis of the treated cells vs. the untreated ones. ^*∗∗∗*^*p* < 0.001 vs. the control (*n* = 2).

To evaluate the changes in the apoptosis rate, Annexin V/PI staining was done in the CT26 cells. The results showed that the apoptosis rate in treated cells was considerably increased (26.64%) versus the control (^*∗∗∗*^*p* < 0.001) (Figure 4).

### 3.5. Treatment with CVC Reduced Mice Tumor Size

**Figure 5:**
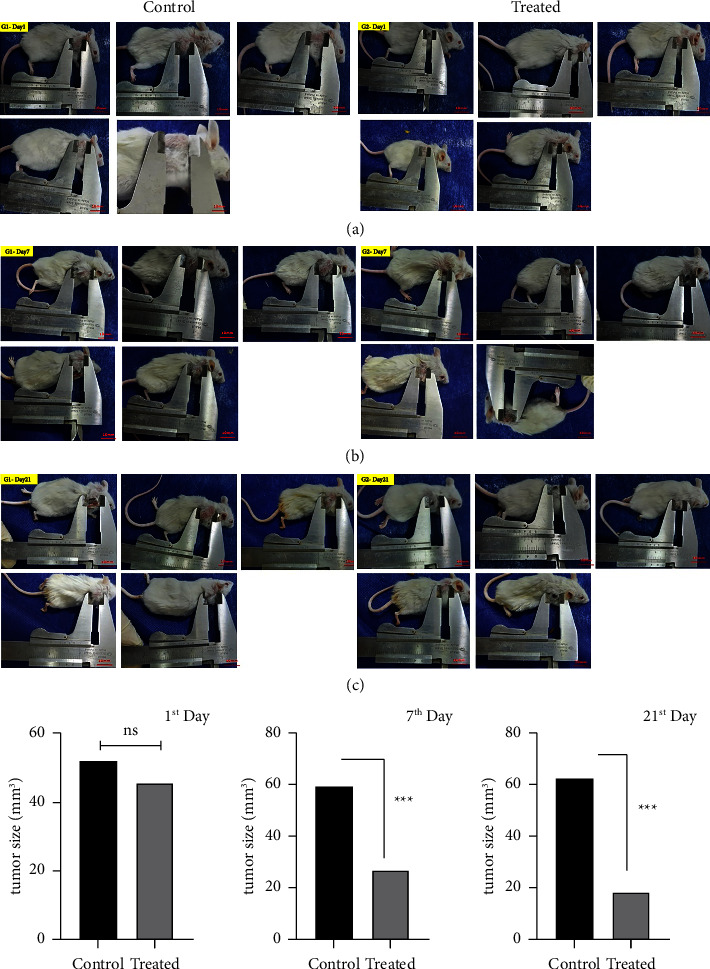
The effect of CVC on the tumor size in the treated mouse model vs. the control group. The size of tumors was measured by a caliper on the 1^st^, 7^th^ and 21^st^ day after treatment. (a) On the 1^st^ day, no significant difference in tumor size was observed in the treated group versus the nontreated group. (b) & (c) CVC reduced tumor size significantly on the 7^th^ and 21^st^ days after treatment. ^*∗∗∗*^*p* < 0.001 vs. the control group (*n* = 2).

To evaluate the effect of CVC on tumor growth, tumor size measurement was done in the treated and control groups on the 1st, 7th, and 21st days after the first treatment. The results obtained from this measurement showed that the tumor size in the treated groups on the first day of injection did not change significantly compared to the control (12.48% reduction) (Figure 5(a)). Nevertheless, on the 7th and 21st day, there was a considerable decrement in the tumor size in the treated mice compared to the untreated ones (54.57% reduction on the 7th day and 70.57% reduction on the 21st day) (Figures 5(b) and 5(c)) (^*∗∗∗*^*p* < 0.001).

### 3.6. CVC Reduced the Expression of CCR2, CCL2, VEGF, Vimentin, c-Myc, NF-*κ*B, and IL33 mRNA Levels in Mouse Models

**Figure 6:**
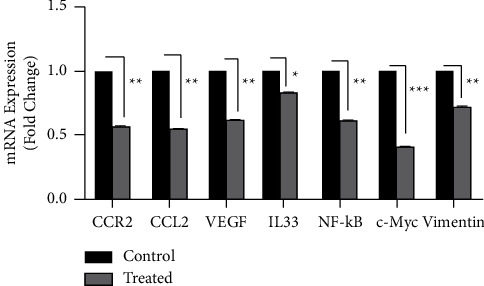
The effect of CVC on the CCR2, CCL2, VEGF, IL33, vimentin, c-myc, and NF-*κ*B gene expression in mouse models. The expression of these genes was dramatically decreased in the treated group ^*∗∗*^*p* < 0.01 and ^*∗∗∗*^*p* < 0.001 compared to the control group (*n* = 2).

The results of qRT-PCR analysis on tumor tissues isolated from mouse models showed that in the treated group, CVC could significantly downregulate mRNA levels of CCR2, CCL2, VEGF, vimentin, c-Myc, and NF-*κ*B, and IL33 compared to the control group (43% reduction for CCR2, 45% for CCL2, 38% for VEGF, 38% for NF-*κ*B, 59% for c-Myc, 28% for vimentin, and 18% for IL33) (Figure 6).

## 4. Discussion

CRC is one of the foremost common neoplasms. Factors such as genetics and environmental factors are effective in the development of this disease [25].

Chemokine signaling can promote cancer progression [2]. Among these chemokines, CCL2, together with its main receptor, CCR2, can increase cancer progression [5]. The expression of this chemokine is increased in CRC [26]. The CCR2_CCL2 signaling pathway significantly increases the survival of cancerous cells, proliferation, and metastasis. This signaling pathway leads to the accumulation of TAMs and MDSCs in the tumor site [5]. TAMs increase malignancy, tumor progression, and angiogenesis and inhibit T cells, which ultimately cause cancer progression and metastasis [9].

MDSCs cause tumor growth through T-cell suppression [4] and also lead to increased angiogenesis and metastasis [27].

Numerous types of research have been done to study the effect of CCR2_CCL2 signaling pathway inhibition in preventing cancer progression [6]. CVC is CCR2 and CCR5 inhibitor [28]. Since CVC inhibits the CCR2_CCL2 signaling pathway, in this research, we used this compound to investigate the effect of CVC on the progression of CRC in mouse models and cell lines.

In this study, the IC_50_ value of CVC was 40 *μ*M, and it was determined by MTT assay, and then, the cells were treated with this concentration in the later steps of the study.

After that, we studied the effect of CVC on the expression of CCR2, CCL2, VEGF, c-Myc, IL33, NF-*κ*B, and vimentin in mRNA and protein levels. Treatment with CVC in CT26 cells dramatically decreased the expression of CCR2, CCL2, and VEGF mRNA levels compared to the control groups in all times points, CCR2 (72% reduction for 24 h, 50% for 48 h, and 31% for 72 h), CCL2 (67% reduction for 24 h, 55% for 48 h, and 66% for 72 h), and VEGF (43% for 24 h, 75% for 48 h, and 73% for 72 h). Also, results showed a significant reduction in mRNA levels of vimentin (22% reduction for 24 h, 50% for 48 h, and 70% for 72 h), c-Myc (90% reduction for 24 h, 71% for 48 h, and 25% for 72 h), NF-*κ*B (42% reduction for 24 h, 61% for 48 h, and 64% for 72 h), and IL33 (60% reduction for 24 h, 50% for 48 and 72 h) in all treated time points. Also, the amount of these proteins in the treated cells had a major reduction compared to the untreated cells (45% reduction for CCR2, 62% for NF-*κ*B, 34% for c-Myc, 55% for vimentin, 44% for IL33, 45.34% for CCL2, and 60.94% for VEGF), at the optimum time. The results of the treatment of animal models showed that CCR2, CCL2, VEGF, vimentin, c-Myc, NF-*κ*B, and IL33 genes expression were reduced dramatically in treated mice compared with the control group (43% reduction for CCR2, 45% for CCL2, 38% for VEGF, 38% for NF-*κ*B, 59% for c-Myc, 28% for vimentin, and 18% for IL33). Since the CCR2_CCL2 signaling pathway can increase the survival of cancer cells, proliferation, and metastasis, CCR2 is considered a potential therapeutic target in cancer treatment [5]. In this context, Gage Brummer et al. reported that CCR2 knockdown can reduce the expression of CCR2 and CCL2 in breast cancer tumors [29]. Also, CCR2 inhibitor can suppress CCL2-mediated lung cancer cell invasion by downregulating MMP-9 expression [30].

The binding of CCL2 to the CCR2 receptor can lead to the activation of several downstream signaling pathways, one of these pathways is the PI3K/AKT/IKK/NF-*κ*B signaling pathway, which can lead to an increase in the expression of CCL2 [31]. Herein, based on our results, we understood that CVC inhibits CCR2, and inhibition of this receptor subsequently can decrease the activity of the PI3K/AKT/IKK/NF-*κ*B signaling pathway, which in turn downregulates the expression of CCL2.

VEGF is a key mediator in angiogenesis [32]. Some researchers have proven that CCL2 can increase angiogenesis by recruiting TAMs and increasing the expression of VEGF in cancer cells [7]. Lien et al. reported that CCL2 caused modulation of VEGF-A expression and angiogenesis through the CCR2/ILK (integrin-linked kinase)/MEK1/2 signaling pathway in OSCC (oral squamous cell carcinoma) [33]. Therefore, it seems that in this study, CVC may reduce the expression of VEGF by affecting CCR2 and its ligand.

Vimentin is considered an important marker for EMT in cancer. It also has an increased expression in several cancers including colorectal [34]. Zhuang et al. reported that the CCL2_CCR2 signaling pathway stimulates invasion and EMT in HCC through the activation of the Hh pathway and up-regulation of Snail and Vimentin. Also, CCR2 silencing causes the inactivation of the Hh pathway, downregulates Snail and Vimentin, and prevents the reduction of E-cadherin [35]. Consequently, it seems that inhibition of the CCR2_CCL2 signaling pathway can downregulate EMT and reduces cancer progression through the inhibition of transcription factors such as Vimentin.

NF-*κ*B signaling is related to the development of CRC [17]. Previous studies have shown that the downregulation of the CCL2_CCR2 signaling pathway by Celecoxib can inhibit the NF-*κ*B pathway [36].

Aberrant expression of c-Myc has been observed in CRC [37]. Teng et al. reported that the inhibition of HCC growth by targeting CCL2 is through the inhibition of STAT3, NF-*κ*B, and c-MYC [38].

IL33 plays a role in many diseases, including cancer. Also, studies have shown that abnormal expression of IL33 has been observed in CRC [39]. Hu et al. reported that IL33 causes the up-regulation of CCL2_CCR2 by activating the NF-*κ*B and extracellular signal-regulated kinase (ERK1/2) signaling in DSCS (decidual stromal cells) [40].

Altogether, we suggested that CVC can reduce NF-*κ*B, c-Myc, and IL33 genes and proteins through inhibition of the CCR2_CCL2 signaling pathway. In addition, as mentioned, one of the signaling pathways activated during the binding of CCL2 to CCR2 is the PI3K/AKT/IKK/NF-*κ*B signaling pathway, which leads to an increase in the expression of CCL2. According to the obtained results, it can be concluded that inhibition of CCR2 by CVC can lead to a decrease in the expression and activity of NF-*κ*B involved in this signaling pathway.

In addition, in this study, the rate of apoptosis in CT26 cells and the effect of CVC on it were investigated by flow cytometry. The results of the analysis showed that CVC could increase the rate of apoptosis in CT26 cells (26.64%). The same result was reported by Lu et al. arguing that treatment of the U251 glioma cell line with CCR2 inhibitor increases apoptosis through the downregulation of phosphorylation levels of p38 and ERK1/2 [41]. In another study, Abd-Rabou and Ahmed showed that the use of CCR2 inhibitor (CR) nanotreatments on the A549 lung cancer cell line increases the apoptosis ratio [42]. Another study demonstrated that CCR2 inhibition improves apoptosis by influencing PI3K/AKT and p38MARK signaling pathway in DLBCL (diffuse large B-cell lymphoma) cell lines such as SUDHL-2 and OCI-Ly8 [43]. The PI3K/AKT and JAK/STAT signaling pathways are downstream pathways that are activated by the binding of CCL2 to CCR2 and lead to the inhibition of apoptosis [6]. Therefore, our findings suggest that inhibition of CCR2 by CVC can lead to a decrease in the activity of these pathways and lead to an increase in apoptosis.

In the animal experiment, we investigated the effect of CVC on tumor size changes in treated mice compared to the control group. Our result showed that CVC reduced tumor size significantly on the 7th and 21st days after treatment (54.57% reduction on the 7th day and 70.57% reduction on the 21st day). In agreement with our result, it has been reported that the knockdown of CCR2 by siRNA inhibits tumor growth in breast cancer [29]. Another study showed that CCR2 antagonist, named 747, can inhibit liver tumor growth by relieving TAMs mediated immunosuppression [44]. Also, the blockade of CCR2 expression reduces tumor growth and inhibits the proliferation of cancer cells by influencing the PI3K/Akt and p38 MAPK signaling pathway in DLBCL (diffuse large B-cell lymphoma) [43]. JAK/STAT and PI3K/AKT signaling pathways are activated during the binding of CCL2 to CCR2, which ultimately leads to an increase in the proliferation of cancer cells [31]. Considering that the proliferation of cancer cells can lead to tumor growth and increase in size, it can be obtained from the results that by inhibiting the CCR2 receptor by CVC, the activity of these signaling pathways is reduced, and subsequently, cell proliferation and tumor size are reduced.

Therefore, taken together, it may be suggested that CVC can be a promising agent to prevent the progression of the tumor through inhibition of the CCR2_CCL2 signaling pathway.

## 5. Conclusion

The main aim of this investigation was to determine the effect of CVC on the main biomarkers involved in the progression of CRC. We demonstrated that this compound can effectively affect these biomarkers and modulate their expression. In addition, it can boost apoptosis and cause tumor growth inhibition. It can be suggested that CVC possesses the potential to effectively modulate the expression of biomarkers downstream of CCR2_CCL2 signaling pathways which are basically linked to the process of invasion and metastasis. Overall, our findings from this research offer that CVC can be considered a promising compound to reduce the progression of CRC through inhibition of the CCR2_CCL2 signaling pathway. Therefore, it should be brought up that this finding provides novel insights into cancer immunotherapy by targeting the tumor microenvironment, and the interactions occurred between tumor cells and cellular and noncellular components. We could present the appropriate influence of CVC action in the prevention of cancer by affecting basic and pivotal targets involved in cancer invasion and metastasis. Further experiments can investigate the CVC effect on biomarkers for cancer cell stemness, EMT, important signaling pathways including PI3K/AKT and JAK/STAT and infiltration and activity of TAMs and MDSCs in tumor microenvironment.

## Figures and Tables

**Table 1:** Primer sequences for real-time PCR.

Gene name	Forward	Reverse
CCR2	CCCTGTCCACTAATGCGTTTCTTATC	TAGCAAAGCCAGACCACAATGAC
CCL2	AGCAGAAGTGGGTTCAGGATTC	TGGGTTGTCGAGTGAGTGTTC
Vimentin	CATTGAGATTGCCACCTAC	CGTTGATAACCTGTCCATC
NF-*κ*B	GGAAGGCAAAGCGAATCCAAAG	CTGTGCGTGGCAACTACATTTC
IL33	AGGTGACGGTGTTGATGGTAAG	AGCTCCACAGAGTGTTCCCTTG
VEGF	GTAACGATGAAGCCCTGGAGT	TGTTCTGTCTTTCTTTGGTCTGC
c-myc	CGGACACACAACGTCTTGGAA	AGGATGTAGGCGGTGGCTTTT
18 s	GTAACCCGTTGAACCCCATT	CCATCCAATCGGTAGTAGCG

## Data Availability

The data used to support the findings of this study are included within the article.
